# Assisted dying requests from people in detention: Psychiatric, ethical, and legal considerations–A literature review

**DOI:** 10.3389/fpsyt.2022.909096

**Published:** 2022-07-29

**Authors:** Irina Franke, Thierry Urwyler, Christian Prüter-Schwarte

**Affiliations:** ^1^Department of Forensic Psychiatry and Psychotherapy, Ulm University, Ulm, Germany; ^2^Psychiatric Services of Grisons, Chur, Switzerland; ^3^Office of Corrections and Rehabilitation, Department of Research and Development, Zurich, Switzerland; ^4^Faculty of Law, University of Lucerne, Lucerne, Switzerland; ^5^Faculty of Law, University of Zurich, Zurich, Switzerland; ^6^Faculty of Medicine and University Hospital Cologne, Institute for the History of Medicine and Medical Ethics, University of Cologne, Cologne, Germany; ^7^Faculty of Health Sciences, Department of Social Philosophy and Ethics in the Health Sciences, University Witten/Herdecke, Witten, Germany; ^8^Department of Forensic Psychiatry and Psychotherapy II, LVR Hospital Cologne, Cologne, Germany

**Keywords:** assisted dying, prison, forensic mental health, severe mental illness, psychological suffering, equality

## Abstract

The principle of equivalence of care states that prisoners must have access to the same standard of health care as the general population. If, as recent court decisions suggest, assisted dying is not limited to people with a terminal physical illness or irremediable suffering, it might also be requested by people with severe mental illness in detention. Some of the countries with legal regulations on assisted dying also have recommendations on how to handle requests from prisoners. However, detention itself can lead to psychological distress and suicidality, so we must consider whether and how people in such settings can make autonomous decisions. Ethical conflicts arise with regard to an individual's free will, right to life, and physical and personal integrity and to the right of a state to inflict punishment. Furthermore, people in prison often receive insufficient mental health care. In this review, we compare different practices for dealing with requests for assisted dying from people in prison and forensic psychiatric facilities and discuss the current ethical and psychiatric issues concerning assisted dying in such settings.

## Introduction

Assisted dying (AD) has always been at the center of heated scientific and public debate. For quite some time, only a few countries (Belgium, Canada, the Netherlands, Luxemburg, and Switzerland) and some states of the United States of America (US) offered legal avenues for AD (1). In recent years, however, numerous jurisdictions have seen a shift toward liberalization (2). In Germany, Austria, and Italy, the constitutional courts repealed or reinterpreted restrictive criminal provisions on AD (3–5), thereby making it (more) accessible, although there is still uncertainty as to how these judgments will be transformed into practice. Furthermore, in 2021 Spain passed a law that legalizes AD (6), and its neighbor, Portugal, is moving in a similar direction, despite objections by the government (7). A similar bill is currently being discussed by the parliament of the United Kingdom (UK) (8). Beyond Europe, both New Zealand (9) and Western Australia (10) have recently implemented laws that make AD available. Recently, Colombia became the first country in South America to allow AD (11), and discussions on AD are emerging also in Asian countries (12–14).

As AD has become more broadly available, medical, ethical, and legal questions have emerged or resurfaced. Many of these questions are difficult to answer, even in case of requests for AD by people not in detention. The situation becomes even more complex if such requests are voiced by people in detention or forensic psychiatric care (henceforth referred to as people in detention, PID). The two main questions are whether legislation allowing AD should include PID, and if so, whether specific aspects must be considered when dealing with AD requests by PID. Answering these questions remains a major challenge. For example, one matter of debate is whether AD should be categorized as a health care intervention (15). If one answers in the affirmative, the principle of equivalence dictates that PID must receive medical care equivalent to that available to the general public (16, 17). Consequently, if AD is available outside prison, it should be available also to PID if their request is well considered and they have sufficient mental capacity (1, 18, 19). And even if one disagrees with characterizing AD as a component of health care, similar conclusions could be reached under the principles of normalization (20) and preservation of human dignity, which necessitate that rights of PID should only be restricted to the extent that is strictly necessary for the purposes of a sentence or measure (15, 21).

When considering this issue, several aspects need to be examined in detail. For example, there is considerable debate as to whether all motives (in particular, mental illness, psychological suffering, and “prison weariness”) can be the basis of legitimate AD requests (22) or whether AD should be limited to people who are suffering because of terminal illness (23–25). If the acceptance of all motives prevails, mentally competent PID could invoke their right to die because of psychological suffering brought about by their mental illness or by the prospect of further deprivation of liberty which, prima facie, seems to be at odds with a state's duty to protect. Detention-specific factors also are relevant in this respect. For example, deprivation of liberty entails a loss of autonomy that raises questions regarding the voluntariness of the wish of PID to end their lives (23, 26, 27). Furthermore, insufficient physical or mental health care in places of detention could directly and indirectly cause PID to want to die (28). In addition, the right to die hinges on someone's mental capacity and on difficult concepts such as unbearable or irremediable suffering, which can be difficult to evaluate (29, 30). Last, in the case of a criminal conviction, AD could create conflicts with the purposes of punishment (22, 23, 31, 32).

The best solution for these issues is far from clear, which is unsettling given the existing demand and support for AD by PID (23). This demand will most likely increase in the future (1, 22) as countries are confronted with aging (prison) populations (33), who will experience the same aging-associated medical conditions as the general population. Furthermore, a relevant proportion of the individuals in preventive detention lacks realistic prospects of release because of the risk of recidivism, which could be an additional catalyst of demand for AD as people become weary of further detention (22, 34). Well-calibrated standards for AD in detention settings clearly are of high importance, considering that life is at stake. However, stable empirical, ethical, and legal standards regarding AD in detention settings have yet to be established (18, 22). The following literature review aims to fill some of this gap and outlines the current empirical, legal, and ethical knowledge on this topic. To this end, we compare current practices for dealing with AD requests from people in prison and forensic psychiatric facilities and point out the emerging, albeit largely unresolved, conflicts.

For the purposes of this manuscript, (preventive) detention comprises deprivation of liberty according to criminal law. The term *forensic psychiatry* refers to all forms of mental health care in secure settings, i.e., prison psychiatry and treatment in secure psychiatric hospitals. Although service structures and legal regulations for referring mentally disordered offenders to secure psychiatric wards or prisons vary between countries, the populations of patients with mental disorders in these two settings seem to be comparable (35, 36). Forensic mental health care comprises the treatment of people who will be discharged after a defined sentence or, in the case of preventive detention, when they are no longer considered dangerous. Regarding AD, different jurisdictions have different understandings of the term (1). In this review, we use AD as an umbrella term that comprises both self-administered AD and health care provider-administered AD (sometimes referred to as “euthanasia”).

## The perspectives of psychiatry, the law, and ethics

### Death and dying in detention

As mentioned in the Introduction, the global prison population is aging (37, 38). Contributing factors include general demographic developments, longer sentences, more restrictive release policies, preventive detention, revolving-door effects, and a higher proportion of older first offenders (39). A prisoner is usually considered to be “old” at an age over 50 years (40), whereas those aged over 80 years are often called the “oldest old” (41). The health status of a 50-year-old prisoner is considered to be equivalent to that of a 60-year-old member of the general population (42). Female older prisoners have been described as particularly vulnerable, considering their status as a “double minority” (being female and old), their health and access to gender-specific health care, and the impact of detention on social relations (43). Although older prisoners are certainly not a homogeneous group, aging usually increases the probability of health-related issues. Consequently, a large number of prisoners are in need of specific care for age-related physical health issues, such as frailty, chronic medical conditions, and polypharmacy and the number will continue to grow. Furthermore, the proportion of age- and disease-related deaths will increase, which will require concepts for palliative care, advance care planning, and—in countries that have respective laws—also access to AD.

These developments pose institutional and personal challenges to representatives of the correctional system. Members of these institutions often see themselves in a custodial rather than a caring role, and the majority of prisoners are young and physically fit men. In case of older prisoners, staff have to confront issues of weakness, illness, death, and dying rather than custody, control, and safety. Some authors argue that de facto life sentences (i.e., longer sentences and less compassionate releases) combined with unhealthy prison environments are a double burden for older prisoners because they subject these prisoners to extra punishment in addition to loss of liberty (41). Handtke and Wangmo (44) conducted a qualitative study with elderly prisoners in Switzerland and found that dying is a relevant topic in this population (for details see next paragraph), but also concluded that death is unwanted or feared in correctional systems because, to some extent, it is seen as a threat to institutional security. In a study from Switzerland, prisoners and stakeholders (prison staff and healthcare professionals, and policy makers), were interviewed, and participants of both groups mentioned AD both for medical reasons and for other reasons, such as indignity and suffering from detention; however some prison staff and policy makers expressed disagreement with AD for prisoners (23). These partly discrepant attitudes of the involved parties illustrate one of the main conflicts in this area.

Prisoners themselves also have to deal with issues of death and dying in detention. In another study from Switzerland, about half of the interviewed older prisoners (total *N* = 35) reported having had thoughts about dying in prison—be it from natural causes or suicide—and about the same proportion admitted to having thought at least once about committing suicide during their detention; in particular, the length of detention and preventive detention seemed to influence how intensively the prisoners thought about their own death (44). Dying in detention usually means dying alone in an uncaring environment (45). With regard to end-of-life care, PID expressed concerns about indifferent health care providers, inadequate medications, delays, and worries about their own physical frailty in the prison environment; at the same time, having a lower perception of one's mental health significantly predicted more fear of death (46). Older prisoners supported the idea of compassionate release for reasons of age and illness, but they also reported contradictory experiences in reality (47). Prisoners treated in specialized facilities for end-of-life care described the care they received as positive, but they also perceived health care needs as being subordinate to security needs (34).

When a prisoner has a terminal illness, one might intuitively assume that risk and security aspects become less important, at least from a medical and humanitarian perspective. However, compassionate release programs—as promoted in several countries—have largely failed to reduce the number of seriously ill prisoners, mainly because the application and review processes are so slow that some prisoners die before completing them (33). Moreover, security concerns may impede the referral of prisoners to hospital and palliative care, not to mention that few requests for referral to palliative care are approved (34).

### Mental disorders and suicidality in detention

Mental health issues are common among PID, and several studies have shown that the prevalence rates of mental and substance use disorders are higher in prisoners than in the general population (48). Among older prisoners, 38% were found to have any psychiatric disorder, which is twice the prevalence reported in the general population; specifically, these prisoners had higher rates of depression, schizophrenia, and anxiety disorders (49). In older prisoners, a diagnosis of posttraumatic stress disorder was associated with impairments in daily life, pain, and poor self-rated health (50). In addition, poor living conditions and overly custodial attitudes of staff can lower quality of life and increase psychological distress in both prisoners and patients in secure psychiatric hospitals (51–53). However, the direction of causality is not clear: the higher prevalence rates may be explained either by direct effects of imprisonment on mental health (54) or by the association of mental disorders with criminal behavior, which in turn increases the likelihood of being imprisoned (55, 56).

This also applies to suicide and self-harm: although it is well established that prisoners represent a high-risk group for self-harm and suicide, it remains unclear whether this association is a result of existing mental disorders, deprivation in detention, or both (57–59). According to a meta-analysis of data from 12 different countries, compared with the general population in most countries, the relative risk of suicide is three to six times higher among male prisoners and over six times higher in female prisoners, although the relative risk in the latter group varies widely (48). A recent meta-analysis identified individual factors (history of or current suicidal ideation, previous self-harm, and psychiatric disorder) and environmental ones (solitary confinement, disciplinary infractions, and victimization) as risk factors for self-harm in male and female prisoners (60). Although considerably fewer data are available for suicide rates in secure (forensic) psychiatric settings, rates probably are quite similar to those in prisoners because the two populations share many suicide risk factors. For example, one study from Germany found no statistically significant difference in the suicide rates in prisons and forensic psychiatric hospitals (61). Suicide risk factors in patients in secure psychiatric hospitals and medium security units overlap to a certain extent with those in the general population, e.g. male sex, severe mental disorder, substance misuse, and previous self-harm (62). In addition, patients in secure settings have specific risk factors, such as long criminal records that include violent or sexual offenses and long sentences (61, 63). Long-term prisoners report more clinically significant symptoms of distress (depressive, paranoid, and psychotic symptoms) than short-term prisoners and have a higher symptom burden than forensic psychiatric inpatients (64).

In accordance with the principle of equivalence of care, PID should receive the same standard of mental health care as any member of the community. The importance of standardized procedures for screening, service provision, training, and professional development has been emphasized, for example by the World Psychiatric Organization (65). Considering the prevalence rates of mental disorders and the risk of suicide in prisons and other secure settings, the provision of adequate mental health care is still in need of improvement (48, 66). Obstacles to sufficient care might be a lack of appropriate interventions, low staff levels, delays to service access, and limited access to training and supervision (67). To date, only one guideline on prison health care for a specific disorder (attention deficit hyperactivity disorder) has been published (68).

In countries where legislation does not restrict access to AD to the presence of a terminal illness (see Table 1), people may also request AD for reasons of irremediable psychological suffering. In the last decade, a few such requests have aroused the interest of the media and the public. One of the most prominent cases was that of a Belgian serial violent and sex offender who had been convicted to a lifelong sentence and was considered as incurable. He applied for AD several times over the course of 3 years. Finally, in September 2014, he was granted the right to decide when to end his life. The decision by the ministry fueled a public debate; one counterargument came from the families of some victims, who argued that this man should not be allowed to evade his punishment. In the end, the ministry revoked its decision in January 2015, mainly because the responsible physician stated that this man was mostly suffering from imprisonment-related pain and therefore tormented by the institution and not by an illness (see, e.g., (26)). This example illustrates almost perfectly the field of tension associated with AD requests from PID.

**Table 1:** Access to assisted dying for people in detention in countries that have decriminalized assisted dying [modified from (1, 2)].

	**Diagnosis/Prognosis**	**Additional assisted dying criteria**	**Assessment procedures**	**Legislation/court ruling specifically referring to people in detention**	**Defined procedures for assisted dying requests by people in detention**	**Data available on assisted dying requests by people in detention**
Austria	Grievous or incurable condition	Majority age, full mental competence	Two assessments (one physician with qualifications in palliative medicine)	Unknown	Unknown	n.a.
Belgium	In adults: incurable condition In minors: terminal condition	Majority age (or emancipated minor); legal competence; voluntariness; well-considered and repeatedly expressed request	Two assessments (third in non-terminal cases) plus committee review	No	Yes	Partially
Germany	N.s. by law	Majority age; full mental competence	Unknown	No. So far, requests by PID have been declined by courts (20)	Unknown	n.a.
Luxemburg	Incurable condition	Majority age; legal competence; voluntariness; well-considered and repeatedly expressed request	Two assessments plus committee review	No	No	No
Spain	Grievous irremediable or chronic condition	Majority age; mental competence; residency in Spain; repeatedly expressed request	Two assessments plus approval by an expert committee	No	No	n.a.
Switzerland	N.s. by law According to a court ruling: severe untreatable physical or psychological condition (69)	N.s. by law According to court ruling (69, 70): majority age; mental competence; well-considered and repeatedly expressed request	N.s. by law. According to medical guidelines: In case of medical conditions that can affect mental capacity, a second assessment is required	Yes (71)	Yes	No
The Netherlands	N.s. by law	Minimum age of 12; voluntariness; mental competence; well-considered request; information about the medical condition and prospects; subjective and professional understanding that there is no other reasonable solution	Two assessments plus committee review	No	No	No
Canada	Grievous and irremediable medical condition (Quebec: serious incurable illness)	Majority age; eligible for national health services; voluntariness; informed consent Mental competence/advanced state of irreversible decline in capabilities	Two assessments	Yes	Yes	Partially
USA^*^	Terminal condition with life expectancy <6 months (Montana: n.s.)	Majority age; mental competence (n.s. in Montana)	Two assessments	No	No	No
Colombia	Terminal condition	Majority age; mental competence	Approval by a multidisciplinary control and evaluation committee	No	No	No
Australia^**^	Terminal condition with life expectancy <6 months (<12 months in neurodegenerative diseases)	Majority age; mental competence (plus detailed state-specific regulations)	Two assessments and committee review	No	No	No
New Zealand	Terminal condition with life expectancy <6 months	Majority age; mental competence	Two assessments (plus competency assessment if required)	No	Unknown	n.a.

*N.s., not specified; n.a., not applicable*.

* *California, Colorado, District of Columbia/Washington D.C., Hawaii, Maine, Montana, New Jersey, New Mexico, Oregon, Vermont, Washington*.

** *South Australia, Victoria, Western Australia*.

### Access to AD for PID in different national legislations

None of the legislations that allows AD explicitly excludes PID or subjects them to a different legal framework for access to AD (1). Therefore, it can be assumed that PID can request AD under the same conditions as members of the community (see also Table 1). However, a few countries have specified procedures for the access of PID to AD, and they monitor the respective requests (1) (see Table 1).

Canada reported a total of 7,595 cases of medical assistance in dying in 2020 (72). Only a very small fraction of these cases are related to PID: As of August 2020, prisoners in Canada had made 11 AD requests, three of which were granted (34). In Belgium, a study from 2015 reported that 17 requests had been made by long-term prisoners who were motivated by the constant and unbearable psychological suffering of detention; all requests were eventually declined (28). In addition, the responsible authorities in these cases had previously refused to make any adjustments to the detention conditions. In the same year according to Belgian authorities, a total of 2022 AD-requests were approved in the general population (73). In Switzerland, results of a self-report study on PID indicated a demand for AD (23), but no official quantitative data on the number of requests and granting/refusals are available. The low prevalence of AD requests by PID, however, must not be taken for a lack of demand. Downie et al. (1) pointed out that no or low rates of requests might not necessarily indicate a low demand but rather that AD programs are not actually available to prisoners.

Canada, Belgium, and Switzerland have regulations or recommendations for the standardized handling of AD requests from PID. In Canada, the request has to be submitted to the institution's health service. Then, the institutional physician or nurse practitioner conducts an initial eligibility assessment with the prisoner. Unlike the general public, prisoners can neither chose the assessor nor ask for a second opinion. If the first assessment is affirmative, a second examination is performed by an external physician or nurse practitioner. If the criteria for AD are met, release options are considered. After a waiting period of 10 days, AD takes place; usually, it is performed at a facility outside the prison, but in exceptional circumstances it can be provided also inside a prison (1). Belgium has a comparable procedure, although only physicians are entitled to assess AD requests (1). In Switzerland, the Swiss Center of Expertise in Prison and Probation (SCEPP) published recommendations on AD in prison settings in 2020 (74). In November 2021, Solothurn—as the first Swiss canton—integrated most of these recommendations into an executive order (71). According to the SCEPP paper, an individual's right to choose the method and time of their own death is not restricted by their detention, as long as their decision-making competency is given. Mental illness and psychological suffering from detention (“prison weariness”) are not mentioned as exclusion criteria. PID who want to end their life have to contact a private AD organization, which evaluates in each specific case whether the legal criteria for AD are met. The recommendations state that internal health professionals and other prison staff should not be asked to participate in the assessments and procedures of AD. The responsible cantonal law enforcement authority has to be informed about the request, is in charge of the further procedure (e.g., collecting medical records) and is responsible also for authorizing temporary absences from prison. In case of considerable doubts about the prisoner's mental competence, the law enforcement agency is entitled to decline the request (74). However, the guidelines are recommendations and do not constitute a law. In addition, the Swiss courts have not yet tried any cases of AD for PID. Thus, the legal framework of AD for PID and associated procedures are still uncertain in Switzerland. It will most likely be the task of the courts to set the legal standards, especially regarding requests for claims of irremediable psychological suffering. Data on AD requests by prisoners are not available. However, a few cases have been reported by the media (e.g., (75)).

### Juridical aspects

#### Duty of care

Giving PID the right to die entails a myriad of legal questions (22, 76, 77) that cannot be addressed fully in this paper. Nevertheless, it is worthwhile to focus on two central aspects, i.e., whether AD for PID is compatible with a state's duty to protect and—in criminal cases—with the purposes of punishment.

The state has a duty to protect the life of people whose liberty it has taken (sometimes also referred to as *duty of care*). As a result, it must intervene if a detained individual attempts to take his or her own life. Not fulfilling this duty would amount to a violation of the right to life, as laid down in Article 2 of the European Convention on Human Rights (ECtHR judgment Miti v. Serbia, January 22, 2013, Section 46; ECtHR judgment People v. Slovenia, December 13, 2012, Section 84; and ECtHR judgment Jeanty v. Belgium, dated March 31, 2020, Sections 70 et seq.). Some scholars have argued that this obligation and the particular vulnerability of PID make AD irreconcilable per se with the state's duty to protect (78, 79). Indeed, deprivation of liberty entails an increase in vulnerability, and the respective individuals are already characterized by a high rate of physical and mental health issues (see section “Mental disorders and suicidality in detention”). Detained individuals often lack support networks, are at an increased risk of physical violence from other PID and lack control over their future (e.g., prospect of release) (31). In addition, the first phase of detention often is accompanied by a surge in suicidal thoughts and behaviors (22). Given this vulnerability, an unconditional right to die for PID would indeed be incompatible with a state's duty to protect (22, 31).

However, the precise legal scope of a state's protective duties regarding the right to life in the context of AD is unclear, particularly in detention settings. Neither the ECtHR nor the supreme courts of the countries where AD is legal have ruled on this topic yet. However, as was pointed out in the Introduction, the principles of normalization and equivalence indicate that once AD is available outside detention settings, it must theoretically be granted inside, too, as long as PID fulfill the general conditions of AD (see Introduction). Given the associated issues discussed above, the law has to put certain safeguards into place (see, e.g., section “Access to AD for PID in different national legistations”), some of which are described by constitutional law and international human right treaties. However, specific cases are lacking. The ECtHR's jurisprudence on AD outside detention settings remarks that states must “establish a procedure capable of ensuring that a decision to end one's life does indeed correspond to the free will of the individual concerned” (ECtHR judgment in Haas v Switzerland, January 20, 2011, Section 58). This principle must apply also in prison settings. The state must confirm the mental capacity of the individual requesting AD (for a discussion of this topic, see section “Access to AD for PID in different national legislations” above). In that regard, there seems to be consensus that unbiased medical professionals who are not connected to the prison system should be charged with the task of evaluating mental capacity (21, 22, 25, 31).

Besides establishing the mental capacity of a detained individual, that individual may not be coerced into making an AD request. Although direct coercion (e.g., pressure on behalf of the prison staff) does not seem to be an issue in practice, as shown by the Canadian experience (34), one could argue that the prison environment is inherently coercive and thus renders a “free” decision impossible (this aspect is both of legal and ethical relevance: see “Ethical aspects”). However, there is reason to assume that such a perspective is too far reaching: as a matter of law, a state must provide detained individuals with adequate physical health care (e.g., palliative care for terminally ill patients) and mental health care and—in the criminal context—with further rehabilitative programs to facilitate reintegration into society (21, 22, 80). If an authority or institution fulfills these conditions and an individual maintains his or her wish to die, AD would be compatible with the state's duty to protect (22). Nevertheless, detention settings do not always live up to the standards of providing sufficient medical care (including palliative care) and release options, among other things (28, 34). For example, if a person is severely ill and his or her physical abilities are substantially reduced, preventive detention could be lifted as the remaining recidivism risk is not high enough to legitimize further detention (20). Regrettably, there is reason to assume that such mechanisms are not used sufficiently, which contributes to suicidality among detained individuals (34).

If all the necessary options are not available or if the available options (e.g., release mechanisms and medical care) are insufficient or insufficiently used, an individual's freedom of choice regarding AD and alternatives may be restricted (1, 34). In these situations, allowing AD in detention settings could resemble an (indirect) death penalty (23, 79), which would violate the state's duty to protect (*indirect* because the structural deficits create the conditions for the individual's motivation to die). At the same time, not allowing AD in situations with structural deficits could be seen equally cynically by the detained individual because the state first brings about the unbearable circumstance through the structural deficits but then refuses to provide access to AD and thereby invokes its duty to protect, the violation of which has given rise to the detainee's wish to die in the first place (22). Thus, although AD does not violate a state's duty to protect per se, it sets very high standards for detention settings regarding psychiatric care and rehabilitative structures, both of which need to exist and be used to the fullest extent (22).

#### Compatibility with the purposes of punishment

Another objection to AD concerns criminal law, namely the compatibility of AD with the purposes of punishment. This point of contention does not apply equally to all penological aims. As regards negative individual prevention in individuals (i.e., deterrence or incapacitation of the offender), AD does not create discordance: If a detained individual ends his or her life, he or she will not commit further crimes (22, 25, 81, 82). Positive individual prevention (i.e., the aim to rehabilitate the offender) requires a more thorough analysis. Some scholars have argued that allowing AD for prisoners is irreconcilable with that aim (25), but this notion has not remained uncontested. Although there is a consensus that the aim of positive individual prevention obliges authorities to provide adequate rehabilitative structures (see above), the rehabilitative aim does not *compel* mentally competent PID to participate in such programs, which is why other scholars consider AD to be reconcilable with such positive individual prevention (22, 31). As far as the aims of negative general prevention (deterrence of the public) and positive general prevention (strengthening society's confidence in the legal system) are concerned, it seems unlikely that public awareness that AD is possible in prison would lead to a relevant increase in crime rates (22, 83), although studies on the impact on crime rates of allowing AD in prison settings do not (yet) exist (25).

The most obvious issue with AD and the aims of punishment concerns the retributive component of punishment (“just deserts”). One could argue that AD gives prisoners the option of evading punishment (or at least a part thereof) by dying before they have served their sentence (22, 23, 31, 32, 81). However, this argument can be made only for people who are serving a criminal sentence. For people in preventive detention, who never had to serve a sentence (e.g., because of criminal insanity), or for offenders who have already served their full sentence and are still being detained because of their recidivism risk, the argument of sentence evasion cannot be entertained (23). As concerns the validity of the evasion hypothesis, some scholars have pointed out that while retribution is relevant for the length of the sentence, its penological importance subsides during the detention phase, where rehabilitation is the dominant principle (22, 31). As a result of this shift, prohibiting AD in prisoners or imposing waiting periods because of retributive concerns would not be proportionate (22, 25). Furthermore, stringent objections have been raised regarding the consistence of the evasion hypothesis as such because it is based on the assumption that PID who die would be better off (32). Because it is impossible to know what follows death, it is also impossible to evaluate whether the individual would benefit more from AD than from staying alive (32). In conclusion, there are strong doubts as to whether retributive concerns are legitimate reasons to restrict access to AD for people serving a criminal sentence.

### Decision-making capacity

As mentioned above, a positive assessment of decision-making capacity is the central precondition for AD in all jurisdictions. Some of the respective laws describe conditions for determining decision-making capacity (84–86). Respect for a patient's self-determined will is both a clinical standard and an obligation required for the initiation of any medical treatment or intervention. In general, most jurisdictions assume that adults are capable of decision-making as long as there is no evidence for any restrictions, e.g., because of a mental disorder. This point is relevant because informed consent to a medical treatment or intervention is only legally and ethically binding if a patient is actually capable of self-determined decision-making. Mental capacity is a multidimensional construct and a central determinant of an individual's ability to make autonomous decisions (85). There is a broad consensus in the literature (86–92) that four elements are related to decisional capacity, i.e., the ability to (1) understand the relevant information, (2) reason rationally, (3) appreciate a situation and its consequences, and (4) communicate a choice (93).

Although decision-making capacity is understood differently in different legal frameworks, there is some consistency in the understanding that the more complex or severe an intervention is and the more enduring its consequences are, the higher are the juridical and medical preconditions for a patient's mental capacity (87, 89, 94). Consent is revocable, and informed consent also implies the ability to disagree. In addition, decision-making capacity is assessed with respect to a defined question, so an individual may be mentally competent regarding certain decisions but incompetent regarding others.

Mental disorders have consistently been assumed to be associated with limited capacity to provide informed consent (84, 88), leading to the question whether there is empirical support for this assumption. In the past few years, a growing number of studies and reviews have considered the decision-making capacity of psychiatric patients, e.g., a systematic review and meta-analysis of factors that help or hinder treatment decision-making capacity in psychosis (95), a systematic review of decision-making capacity for treatment and research in patients with schizophrenia and other non-affective psychoses (96), a systematic review of lack of mental capacity in psychiatric and medical settings (97), and a more general review of clinical and epidemiological factors that have an impact on mental capacity in the psychiatric population (85). The review by Okai et al. (85) reports that incapacity is common among psychiatric inpatients (in a median of 29%) but that the majority are capable of making treatment decisions. Psychosis, severe symptoms, involuntary admission, and treatment refusal were reported as the strongest risk factors for incapacity in psychiatric patients. Other studies found that cognitive symptoms in patients with schizophrenia (90, 91) or a lack of disease insight in psychotic patients (88) are more significant risk factors for mental incapacity than psychopathological symptoms.

Most studies agree that the decision-making capacity of people with a severe mental disorder can be improved or re-established by improving cognition, for example by cognitive remediation or repeating information (86, 92).

On the other hand, empirical evidence exists showing that the majority of psychiatric patients are actually competent to make decisions. A recently published meta-review found that up to 75% of the assessed psychiatric patients were mentally competent at the time of assessment or were able to achieve mental competence during treatment (92). According to another review of 33 studies (86), the prevalence of decision-making capacity varied between 5 and 83%: it ranged from 7.7 to 42% in patients in involuntary treatment and from 29 to 97.9% in patients in voluntary treatment. However, both sets of authors reported that all studies in their review excluded forensic-psychiatric patients, although they did not explain why. Presumably, the high obstacles for performing research in this population (see section “Ethical aspects”) explain this exclusion. Therefore, it seems important to analyze the extent to which the results can be replicated in forensic settings.

Besides the general discussion about decision-making capacity in people with severe mental illness, a further question is how it should be assessed. In clinical practice, decision-making capacity is usually determined by a psychiatrist on the basis of his or her personal evaluation and clinical experience (84, 98). To provide a more objective and reliable evaluation, in recent years several structured assessments based on different ethical and legal concepts were developed [for a review, see Palmer and Harmell (89), McSwiggan et al. (94), Bauer and Vollmann (99)]. The MacArthur Competence Assessment Tool-Treatment (MacCAT-T) is widely used in clinical psychiatry (90). It is a semi-structured interview that requires 20–25 min to complete (100) and assesses different components of decision-making capacity on four subscales (understanding, appreciation, reasoning, and expression of a choice). The MacCAT-T has high objectivity, validity, and reliability (89). However, it operationalizes all four dimensions of decision-making capacity mainly by cognitive and intellectual abilities and does not question whether decision-making situations are exclusively based upon rational choices or consider emotionality as an adequate and useful tool for decision-making strategies. Consequently, additional assessments were developed in recent years (101). Another critical aspect is that people with mental disorders more often were considered to be incompetent when assessed with the MacCAT-T than with a clinical evaluation (90). Vollmann (102) indicated that insufficient reliability in the assessment of decision-making capacity may lead to a high probability of error, i.e., some people are illegitimately deprived of their right to decide, whereas others with probably more severe impairment are falsely considered as competent. From an ethical perspective, it is preferable that an incompetent patient is considered as competent (false positive) rather than vice versa (false negative). Considering this argumentation and the results of empirical studies (89, 94), the established clinical evaluation seems to be preferable to the exclusive use of structured assessments. An integrative approach combining clinical and structured assessments might improve the results; however, further research is needed.

### Ethical aspects

The essential ethical questions regarding this paper's topic can be summarized as follows: Are PID (either in prisons, preventive detention, or secure psychiatric hospitals) autonomous and able to make self-determined decisions? What if they also have a mental disorder that permanently affects their cognition and volition and thereby their ability to make autonomous decisions? In the following section, these ethical questions are discussed taking into account the specific situation of PID. The fundamental question of whether AD can be justified in general would go beyond the scope of this article, and will therefore not be discussed here. In the context of imprisonment and secure treatment settings, self-determination is the central ethical issue.

PID live in environments described as *total institutions* by Goffman (103). These institutions have the power to regulate and control literally every aspect of an individual's life (104). Several scholars have repeatedly expressed considerable doubts regarding the extent of autonomy of PID (105), as can be seen for example in the discussion about the legitimation of research in this population. Not only because of the effects of detention in such institutions, but also in response to the historic crimes committed in the name of medical research during National Socialism in Germany (106, 107), medical research ethics define PID and psychiatric patients as a particularly vulnerable group (108). The vulnerability of mentally disordered offenders in prisons or secure psychiatric settings extends to at least two levels: “... detained Mentally Disordered Offenders are especially vulnerable, both since they are placed in a coercive institutional context where they to a large extent have to depend on the good will of its representatives and since their health status often has negative effects on their ability to protect and further their own interest.” (109). Therefore, in many countries research on PID is substantially restricted, if not forbidden. The German Medicines Act (section 40) categorically bans pharmaceutical research on PID and bases this ban on the argumentation that, in the context of “characteristic relations of power” in custodial institutions, the necessary freedom to autonomously decide for or against research participation is irrefutably missing (110). This juridical evaluation has led to a generally negative attitude of ethic committees toward research in forensic psychiatry in Germany (111, 112). Besides the restriction of civil rights and the impairments caused by the mental disorder itself, additional, more subtle forms of coercion and manipulation that are especially relevant under conditions of deprived liberty have been brought forward as arguments (113).

In most countries' legislation, referral of mentally disordered offenders to (secure) psychiatric hospitals is based on diminished criminal responsibility. Psychopathological symptoms that affect criminal responsibility are often identical with those that affect autonomous decision-making. Therefore, the referral itself to secure psychiatric settings might raise general doubts about the autonomy of this subgroup of PID. Such doubts about autonomy are essential for the topic of this paper because authors who support AD for mentally disordered persons underline the importance of autonomy and decision-making capacity as preconditions for granting such requests (114–116). As mentioned above (Table 1), national legislations explicitly demand sufficient mental competence and informed consent as necessary conditions for AD.

There is broad consensus in the literature that numerous psychiatric disorders may at least temporarily affect the ability to realize personal autonomy in daily life (117). However, it should be critically noted that the terms *autonomy* and *independence* are often used interchangeably (118), irrespective of their theoretical background (117). The term *autonomy*, which in many medical situations is usually bound to the sphere of abstract reflection or inconsiderate practice and not affected by the empirical urge to act, remains shallow and non-binding—and may become a risk for the affected patient. This risk is of special relevance if the term is used uncritically in psychiatric settings with the consequence of leaving suffering individuals on their own (119).

On the other hand, a categorial denial of decision-making capacity in psychiatric patients does not withstand a critical evaluation and might even be considered as stigmatization. Consequently, research does not support a categorical denial of mental competence in these patients, as described above (see section “Decision-making capacity”). Thus, in countries that have respective legislation allowing AD, individuals with mental disorders cannot be generally refused access to AD (120). This statement applies also to PID with mental disorders in prisons or secure psychiatric hospitals. Nevertheless, even if mental competence is given, the above-mentioned doubts persist as to whether and how far the specific institutional context has a negative impact on the free will and autonomous decision-making of PID. In addition, the fact that both the above-mentioned reviews on mental competence (86, 92) excluded forensic-psychiatric patients illustrates the doubts that still exist in many jurisdictions regarding the autonomous decision-making capacity of PID. Besides the characteristics of total institutions, the substandard provision of mental health care and end-of-life care, as well as the lack of perspectives for long-term prisoners, further contribute to these doubts (121, 122). Such environmental factors can increase suicidality by evoking feelings of futility and lack of prospect (see also “Mental disorders and suicidality in detention”).

Recent publications have referred to the role of the concept of meaningfulness for the ethical evaluation of AD (123, 124). According to the authors of these papers, suicide can be interpreted as a negation of meaningfulness and ought to be counteracted by society. Public institutions such as prisons and psychiatric hospitals are not excluded from this responsibility because it is one of their duties to enable a meaningful life within these institutions, especially for PID in long-term detention. From an ethical perspective, this might also be an answer to AD requests from PID.

In conclusion, doubts remain as to whether autonomous AD requests are actually possible under the described living conditions.

## Discussion

Given the changes over the last two decades in AD legislation, and in particular the obviously more liberal public and political attitudes (expressed, e.g., by the German constitutional court's decision in 2020), AD is inevitably going to be considered and (in some cases) requested by PID for various reasons, such as terminal physical illness, a persistent and irremediable mental disorder, or psychological suffering from detention (“prison weariness”) (15).

AD requests from prisoners with a terminal physical condition pose different legal and ethical conflicts at least to a certain degree, than requests from those who claim psychological suffering or who have a mental disorder. If a country decriminalizes AD for the terminally ill, the principle of equivalence (if AD is considered as a medical intervention) or normalization (if AD is not considered as a medical intervention) dictates that PID cannot be excluded; consequently, feasible processes are required for requests, assessments, and procedures that safeguard autonomy and, at the same time, protect PID from themselves if they lack mental competence. Considering the limited resources for specialized end-of-life or palliative care in custodial settings (34), it seems vital to emphasize that AD should not become the more easily available option by which PID can maintain self-determination. Therefore, detention settings must provide adequate structures that give an individual the full range of options.

The debate on AD for PID with mental disorders and those who claim unbearable psychological suffering from detention is much more complex. Given the prevalence rates of mental disorders in prison settings (48) and the potentially adverse effects of long-term detention on mental health (52, 58), PID undoubtedly constitute a vulnerable population in multiple respects and consequently require special protection.

In case of AD for reasons of constant and irremediable suffering from a mental disorder, beyond the well-known difficulties of determining irremediable suffering and the terminal character of mental disorders (120), one must consider also the possibility of insufficient provision of mental health care. In this respect, the principle of equivalence of care for PID has at least two dimensions: equivalent access to AD on the one hand, but also equivalent access to mental health services or psychiatric inpatient treatment, if necessary, on the other. From a juridical perspective, these two qualities of equivalence may not be hierarchical, but from a medical perspective, they can hardly be supported if limited resources for treatment in secure settings result in suicidality and AD requests. However, this issue is also fluid in nature because the definition of adequate mental health care is a matter of constant debate and also depends on subjective factors. Consequently, care levels may be seen as sufficient on an objective level but as insufficient by an individual.

In addition, evaluating decision-making capacity is challenging even in non-custodial situations. Although decision-making capacity is usually not given in mental states with severe misperceptions of reality and self, such as advanced dementia, schizophrenia, and major depressive disorder (85, 87, 88, 90, 91, 94), each case has to be evaluated diligently because symptoms of most mental disorders fluctuate (as does decision-making capacity in people with mental disorders) (22) (see section “Decision-making capacity”). As discussed above, structured assessments can assist evaluations of mental capacity but should be used in addition to clinical evaluations (93). Furthermore, some research has examined the decision-making capacity of psychiatric patients: Findings indicate that mental capacity for treatment decisions is given in the majority of cases but that symptom severity and a lack of disease insight are more likely to be associated with incapacity (85, 86, 92). However, the discussion about autonomy and decision-making capacity still is a more or less theoretical or academic one. To the authors' knowledge, no studies have addressed these questions in actual PID who request AD, which is not surprising, given the relative novelty of the topic. Although it is methodologically and legally challenging (e.g. ethical approval etc.), research is urgently needed on voluntariness and decision-making capacity of PID.

In conclusion, from a legal perspective (principles of equivalence and normalization), identical AD criteria should be defined for PID and the general population, as long as no specific reasons exist why we should deviate from that standard. However, in legislations that do not exclude people with a mental disorder from access to AD, strong safeguards are necessary to prevent a slippery slope toward AD becoming the preferred method for ending suffering from untreated or incurable mental disorders in detention because other options (e.g., sufficient mental health care) are lacking. When considering the duty of the state to protect and the duty of health professionals to avoid harm for the patient, we must appropriately address the state of vulnerability of PID when processing AD requests. Therefore, assessments of PID who request AD should pay attention to the following aspects: (1) careful evaluation of the current mental state, including the role of external factors (e.g., conditional release was recently declined, person was transferred to another institution or victimized); (2) evaluation of the past and current treatment options available in detention compared with those available in the general community; and (3) provision of at least two independent assessments of mental competence by physicians familiar with both forensic/prison psychiatry and palliative medicine. In addition, waiting periods should be discussed in all non-terminal conditions (21).

AD requests from PID because of psychological suffering from detention are different from the other two types of requests discussed above because such people do not necessarily have an underlying medical condition: The reason for the psychological suffering is mainly the detention itself and/or the resulting living conditions. The existing research indicates that there is an association between detention conditions, the psychological state, and the will to live or die (53, 57). This does not mean that psychological suffering from detention is less severe than that from physical or mental disorders, especially because individual suffering can only be quantified by an individual him- or herself with their own values, life plans, etc. and is impossible to quantify objectively. However, it does mean that psychological suffering from detention might be alleviated by changing the circumstances of detention (see also below). The unresolved moral and juridical challenge of such requests is well illustrated by the fact that, to date, no legislation has granted AD to PID because of psychological suffering alone (1, 28). With respect to recent developments, e.g., in the jurisdiction in Germany, motives for requesting AD might become more or less relevant in the near future if self-determined dying is legally defined as the personal right of every mature and competent person irrespective of the presence of a terminal illness or irremediable suffering. Under such preconditions, and once there is agreement that PID are capable of autonomous decisions, mental competence/informed consent would become the only crucial medical factor in the evaluation of AD requests or treatment refusals.

In this context, one must remember that AD is not (yet) a general global phenomenon but more or less a development in individualistic and less religious societies. As has been shown, e.g., by data from the US, and is also illustrated by the countries that have decriminalized AD, it is mainly used by people with a stable economic-educational background (125). In contrast, PID often have a poor socioeconomic status and come from a variety of cultural backgrounds with a variety of moral values.

Both imprisonment and treatment in secure forensic psychiatric settings are associated with considerable and long-lasting restrictions of an individual's personal freedom, especially when someone is considered as dangerous and a persistent risk to public safety. Many of the prisoners in life-long detention have mental disorders, especially personality disorders, that have been deemed as untreatable and/or incurable. Because risk-orientated detention is unlikely to become less frequent in criminal policy in the near future, we need to reconsider the living conditions of PID with no prospects of release in the foreseeable future and to discuss alternatives to highly restrictive forms of preventive detention [e.g., see the discussion in Germany on the so-called “Abstandsgebot” for people in indefinite preventive detention (126)]. However, preventive detention depends to a large extent on societal and political attitudes and values. Some authors put the current developments in a wider context, arguing that the rising numbers of prisoners and preventive detentions are the result of a neoliberal management of poverty in which crime is seen as the failure of an individual rather than a structural societal issue (127, 128). Such a development would undermine the concepts of not only offender rehabilitation, but also autonomy, humanity, and solidarity. Thus, utmost efforts need to be undertaken to hold conditions in detention settings to very high standards and use all available measures to rehabilitate and reintegrate PID. These steps seem to be the best means to ensure that PID and the detention system see AD as an exception that is to be used with great caution.

## Author contributions

IF wrote section Death and dying in detention and Mental disorders and suicidality in detention. IF and TU wrote section Access to AD for PID in different national legislations. TU wrote section Juridical aspects. CP-S wrote sections Decision-making capacity and Ethical aspects. All authors contributed to the conception of the review, the introduction and the discussion section and contributed to manuscript revision and read and approved the submitted version.

## Conflict of interest

The authors declare that the research was conducted in the absence of any commercial or financial relationships that could be construed as a potential conflict of interest.

## Publisher's note

All claims expressed in this article are solely those of the authors and do not necessarily represent those of their affiliated organizations, or those of the publisher, the editors and the reviewers. Any product that may be evaluated in this article, or claim that may be made by its manufacturer, is not guaranteed or endorsed by the publisher.
